# Effects of Fermented Forage-Based Mixed Feed on Growth Performance and Meat Quality in Finishing Pigs

**DOI:** 10.3390/vetsci13080757

**Published:** 2026-07-30

**Authors:** Baorui Liu, Guoqiang Cui, Haiyan Xiao, Zhiqiang Peng, Jianguo Tan, Xianren Jiang, Kun Meng

**Affiliations:** 1 Key Laboratory of Feed Biotechnology of Ministry of Agriculture and Rural Affairs, Institute of Feed Research, Chinese Academy of Agricultural Sciences, Beijing 100081, China; liubr2001@163.com; 2 Changsha Runfeng Ecological Agriculture Technology Development Co., Ltd., Changsha 410600, China; cuiguoqiang100@126.com (G.C.); pengzhiqiang1977@126.com (Z.P.); 3 Agriculture and Rural Affairs Bureau of Ningxiang City, Ningxiang 410600, China; xiaohaiyan1006@126.com; 4 Hunan Kangyongda Ecological Agriculture Co., Ltd., Changsha 411200, China; tanjianguo1016@126.com

**Keywords:** fermented forage-based mixed feed, finishing pigs, growth performance, meat quality

## Abstract

This study aimed to assess the effects of replacing 20% of the standard diet with fermented forage-based mixed feed (FF) on finishing pigs. The *Pennisetum No. 1* forage powder, feed ingredients, and composite microbial inoculum were mixed, and the mixture was incubated for 7 days at room temperature, maintained above 25 °C. A total of 176 finishing pigs were randomly assigned to two dietary treatments: a basal diet (CON) and an experimental diet with 20% of the basal diet substituted by FF (20% FF). Each treatment consisted of four replicates, with each replicate including 22 pigs. Throughout the 56-day trial, the findings showed that there were no notable differences (*p* > 0.05) in growth performance, apparent nutrient digestibility, carcass characteristics, and muscle fatty acid profiles between the control group and the 20% FF group. However, the dietary inclusion of 20% FF significantly enhanced glutathione peroxidase (GSH-Px) activity by 46.59% and decreased the malondialdehyde (MDA) concentration by 22.79% in the Longissimus thoracis et lumborum muscle (*p* < 0.05), suggesting enhanced muscular antioxidant capacity. In summary, substituting 20% of the basal diet with FF represents a feasible approach that may improve the antioxidant capacity of pork while sustaining both growth performance and carcass characteristics in pigs. This research establishes a scientific foundation for utilizing FF as a practical alternative feed resource in finishing pig production.

## Simple Summary

To mitigate escalating swine feed costs and the over-reliance on imported raw materials, this research explored the potential of utilizing fermented forage-based mixed feed (FF) as an alternative dietary (as-fed) ingredient for finishing pigs. A feeding trial lasting 56 days was carried out in which 20% of the base diet was substituted directly with FF. The results indicated that this substitution strategy exerted no adverse effects on the growth performance or meat quality of the pigs. Importantly, the inclusion of this diet markedly improved the antioxidant properties of the pork. By utilizing local forage resources, this approach provides a practical alternative strategy to reduce reliance on conventional feed ingredients in swine production.

## 1. Introduction

In China, the swine industry relies heavily on imported feed resources, particularly soybeans and corn, making feed costs highly susceptible to international market fluctuations; notably, in 2025, soybean imports climbed to an unprecedented 111.83 million tonnes, with an import reliance surpassing 80% [1,2]. In response to the policy of “reducing and substituting soybean meal,” developing and utilizing localized, non-conventional forage resources has emerged as a strategic priority. *Pennisetum*, a premium perennial grass, is commonly found in tropical areas, featuring high yield, robust adaptability, and excellent palatability [3,4]. However, its direct application in monogastric diets often reduces voluntary feed consumption, mainly as a result of its elevated lignocellulose levels and the existence of anti-nutritional components [5].

Microbial fermentation technology offers a highly promising solution to overcome the limitations associated with the application of *Pennisetum No. 1* forage grass in monogastric diets. During fermentation, endogenous enzymes naturally present in the substrate (e.g., endogenous phytase) act synergistically with enzymes produced by fermentative microorganisms to degrade structural carbohydrates and anti-nutritional components in feedstuffs, resulting in the production of various bioactive compounds [6,7,8]. Research conducted previously has shown that fermented forages, including alfalfa and leaves of the mulberry plant, can greatly enhance intestinal health and antioxidant abilities in swine [9,10]. Furthermore, supplementing diets with fermented mixed feed or fermented pine needles has been proven to optimize meat quality in finishing pigs [11,12]. In southern China, many small- and medium-scale pig farms have access to locally available forage resources and other plant-based feed materials, which can be utilized as alternative feed ingredients [13]. Combining locally adaptable forage resources with microbial fermentation technology can convert fresh forage into a nutritionally rich fermented feed with stable quality, thereby reducing reliance on commercial feeds and lowering overall feed costs [14].

Notably, most previous studies have evaluated fermented feed under isoenergetic and isonitrogenous dietary conditions, whereas commercial pig production often adopts a direct replacement strategy to reduce feed costs without reformulating the entire diet. Furthermore, systematic evaluations of forage-based fermented feeds in finishing pigs remain limited, with a particular lack of sufficient data regarding the optimal replacement ratio. Therefore, this study utilized *Pennisetum No. 1* forage grass as the primary substrate to prepare FF via microbial fermentation. By replacing 20% of the basal diet with FF, we conducted a systematic assessment of its impact on growth performance and meat quality. The objective of this research was to establish a scientific foundation for applying FF in the production of finishing pigs, as well as to deliver both theoretical insights and practical assistance for creating a cost-effective, eco-friendly model for feeding.

## 2. Materials and Methods

### 2.1. Experimental Materials

*Pennisetum No. 1* forage grass was sourced from the Changsha Runfeng Company. Cornmeal suitable for feed, feed-grade wheat bran, and soybean meal were obtained from a nearby market. The specific fermentation inoculum for feed utilized in this research was provided by Zhongke Jiayi Bioengineering Co., Ltd., located in Shandong, China. The commercial inoculum was a composite microbial inoculant containing *Lactobacillus plantarum*, *Pediococcus pentosaceus*, *Lactobacillus buchneri*, *Lactobacillus casei*, and *Saccharomyces cerevisiae*. The inoculant was applied according to the manufacturer’s recommended dosage, resulting in an initial inoculation level of approximately 1.0 × 10^9^ CFU/kg of substrate for each microbial species.

### 2.2. Preparation of Fermented Forage-Based Mixed Feed

Forage exceeding 3 m in height was ground into particles shorter than 3 mm. The four components were combined in a mixer based on the proportions outlined in Table 1. During the mixing process, the microbial inoculum was applied uniformly and gradually, with the initial moisture content of the mixture being modified to around 43%. The breathable fermentation bags were each filled with a mixture weighing 25 kg, ensuring sufficient air space was preserved. The bags were moved to a fermentation chamber where they were incubated for a week at a room temperature kept above 25 °C.

### 2.3. Location and Timeline of Animal Experiments

The experiment involving animals took place in a commercial pasture in Ningxiang, Hunan Province, China, spanning from November 2024 through January 2025. Approval and supervision for the animal experiments were granted by the Animal Welfare and Ethics Committee at the Institute of Feed Research, Chinese Academy of Agricultural Sciences (Approval No. IFR-CAAS-20241118).

### 2.4. Experimental Design and Animal Husbandry

Briefly, 176 healthy growing-finishing barrows (with an initial average body weight of 65.80 ± 1.66 kg, all crossbred individuals of Duroc, Landrace, and Yorkshire (DLY) breeds) were randomly divided into two groups based on body weight (BW) for dietary treatments. Each treatment had four replicate groups, and each replicate group contained 22 pigs. The feeding treatments consisted of a control diet (CON) and a diet in which 20% of the control diet was substituted with FF on an as-fed basis (20% FF). The duration of the experimental feeding phase was 56 days.

The foundational diet designed for pigs was prepared in accordance with the nutritional needs outlined by the NRC (2012) for pigs. Table 2 displays the nutritional composition and levels of this foundational diet. Prior to animal placement, the pens and experimental equipment were thoroughly cleaned, disinfected, and properly ventilated to dry. All pigs were housed in the same facility, with each replicate assigned to a single pen (3.0 m × 7.5 m) containing 22 pigs. The pens had concrete floors with partial slatting and were furnished with a feeder and a drinker, enabling continuous access to food and water.

### 2.5. Measurements and Methodologies

#### 2.5.1. Chemical Analysis

All feed and fecal samples were assayed for routine nutrients in strict accordance with official standard methods. Specifically, dry matter (DM, Method 930.15), crude protein (CP, Method 984.13), ether extract (EE, Method 920.39), and crude ash (Ash, Method 942.05) were determined following the procedures specified in AOAC Official Methods of Analysis [15]. The contents of crude fiber (CF), neutral detergent fiber (NDF), and acid detergent fiber (ADF) were measured with an ANKOM fiber analyzer (ANKOM Technology, Macedon, NY, USA) referring to the classic Van Soest detergent fiber method [16]. The gross energy (GE) of each sample was evaluated using a Parr 6400 adiabatic oxygen bomb calorimeter (Parr Instrument Co., Moline, IL, USA). Determinations of acid-insoluble ash (AIA), calcium (Ca), and phosphorus (P) were carried out following the guidelines of GB/T 23742-2009 [17], GB/T 13885-2017 [18], and GB/T 6437-2018 [19]. Lysine, methionine, and threonine were analyzed in accordance with GB/T 18246-2019 [20]. The assessment of tryptophan was carried out following GB/T 15400-2018 [21].

The pH level of the FF was assessed utilizing a multifunctional meter known as the SevenMulti (Mettler-Toledo, Greifensee, Switzerland). The analysis of organic acid concentrations, including lactic, acetic, propionic, and butyric acids, was conducted using a Metrohm 940 professional ion chromatography system (Metrohm AG, Herisau, Switzerland), which was outfitted with a Metrosep Organic Acids-250/7.8 ion-exchange column and an MSM suppressor. The separation process was carried out with 0.50 mmol/L H_2_SO_4_ serving as the mobile phase in an isocratic elution at a flow rate of 0.60 mL/min. The column temperature was controlled at 30 °C, the sample injection volume was set to 20 μL, and a 100 mmol/L lithium chloride solution was used for suppressor regeneration.

The viable populations of lactic acid bacteria (LAB) and yeast in FF were assessed in accordance with GB/T 34224-2017 [22]. LAB were enumerated on de Man, Rogosa, and Sharpe (MRS) agar medium, while yeasts were cultivated on yeast extract peptone dextrose (YPD) agar. All microbial populations were quantified by the standard pour plate counting method, and results are expressed as log_10_ colony-forming units per gram of fresh sample (log_10_ CFU/g).

The concentrations of aflatoxin B_1_ (AFB_1_), zearalenone (ZEN), ochratoxin A (OTA), and deoxynivalenol (DON) in FF were determined on a DM basis. AFB_1_ and ZEN were assayed in accordance with the Chinese agricultural industry standard NY/T 2071-2011 [23], whereas OTA and DON were determined according to GB/T 30957-2014 [24] and GB/T 30956-2014 [25], respectively. The results are expressed as mg/kg DM.

#### 2.5.2. Growth Performance Calculation

Daily feed provisions for each pen were documented, and unconsumed feed was weighed on the mornings of days 28 and 56 to determine intake. Each pig in the pens was weighed individually on the mornings of days 0, 28, and 56 and throughout the trial.

Growth performance was evaluated by three core indices: average daily gain (ADG), average daily feed intake (ADFI), and feed-to-gain ratio (F/G), all calculated based on body weight and feed intake data.

#### 2.5.3. Apparent Nutrient Digestibility

On the 56th day, rectal massage was employed to collect fecal samples from 2 to 3 pigs in each pen, which were then combined to reach a total weight of around 200 g. These samples were subsequently dried for 48 h at a temperature of 65 °C, then crushed and passed through a sieve measuring 0.425 mm. Samples were kept at 4 °C pending additional analysis. The apparent total tract digestibility (ATTD) of GE, DM, CP, and crude ash was determined by the endogenous indicator method, with AIA selected as the internal marker.

The calculation of apparent nutrient digestibility (%) can be represented by the following formula: [1 − (*A*_1_ × *F*_2_)/(*A*_2_ × *F*_1_)] × 100. Here, *F*_1_ and *F*_2_ represent the nutrient concentrations in diet and feces, respectively; *A*_1_ and *A*_2_ denote the AIA concentrations in diet and feces, respectively.

#### 2.5.4. Carcass Traits

On day 56, one pig per pen with body weight nearest to the pen average was chosen following a 12 h fast and then electrically stunned and bled out.

Following slaughter, the carcasses were sliced longitudinally along the dorsal midline. The left half of the carcass was used to assess the mean thickness of backfat and to obtain the *Longissimus thoracis et lumborum* muscle. Carcass parameters, including dressing percentage, average backfat thickness, and lean meat percentage, were evaluated in accordance with previously established protocols [12,26]. The left half-carcass was used for average backfat thickness measurement and Longissimus thoracis et lumborum muscle sampling.

#### 2.5.5. Meat Quality Assessment

Portions of the *Longissimus thoracis et lumborum*, located between the left side’s third and fourth-to-last ribs, were rapidly removed during the processing stage. These samples were subsequently deposited into cryovials, snap-frozen with liquid nitrogen, and transferred to an −80 °C ultra-low temperature freezer for future analysis.

The parameters of meat color, which encompass lightness (L*), redness (a*), and yellowness (b*), were assessed 45 min after death with a CR-10 Chroma Meter (Konica Minolta, Osaka, Japan). Before conducting the measurements, the device was calibrated using the standard white calibration plate provided by the manufacturer, following the specified operating guidelines. Readings were conducted at various locations, ensuring the exclusion of apparent fat and connective tissue. The assessment of antioxidant parameters, including MDA levels and the activities of enzymes like catalase (CAT), glutathione peroxidase (GSH-Px), and superoxide dismutase (SOD), was performed using commercial assay kits (Nanjing Jiancheng Bioengineering Institute, Nanjing, China), with all operations strictly following the kit instructions. The profile of fatty acids in the muscle was analyzed using gas chromatography (Agilent 6890 series, Wilmington, DE, USA), following the methods outlined by Liu et al. [11]. Fatty acid methyl esters (FAMEs) were isolated utilizing an HP-88 fused-silica capillary column (Agilent Technologies, Santa Clara, CA, USA). A carrier gas of high-purity nitrogen was used, with a consistent flow rate of 1.0 mL/min. A volume of 1 μL of the sample was injected in split mode, utilizing a split ratio of 50:1. The injector’s temperature and the flame ionization detector (FID) were set at 250 °C and 260 °C, respectively. The oven’s temperature program was organized as follows: it started at 140 °C for a duration of 5 min, then increased to 220 °C at a rate of 4 °C/min, and was held at 220 °C for a further 15 min.

### 2.6. Statistical Analysis

All experimental data underwent statistical evaluation using the General Linear Model (GLM) approach in SAS 9.4 software (SAS Institute Inc., Cary, NC, USA). The statistical model included dietary treatment as the fixed effect:

*Y_ij_ = μ + T_i_ + e_ij_*

where *Y_ij_* represents the observed value of each test index, *μ* is the overall population mean, *T_i_* stands for the fixed effect of the *i*-th dietary treatment (*i* = 1, 2, corresponding to the CON group and 20% FF group, respectively), and *e_ij_* is the random residual error. Normality and homogeneity of variance were checked prior to analysis. For growth performance and nutrient digestibility indices, each pen was regarded as one independent experimental unit; for carcass traits and meat quality indices, each slaughtered individual pig was taken as the experimental unit. Since only two treatment groups were set in this trial, an independent-sample *t*-test was adopted for inter-group comparison on the basis of GLM analysis. All results are expressed as mean ± standard error of the mean (SEM), and *p* < 0.05 was defined as the threshold for statistically significant difference.

## 3. Results

### 3.1. Fermentation Characteristics and Nutritional Composition of the Fermented Forage-Based Mixed Feed

Following seven days of anaerobic fermentation, the resulting FF exhibited a yellowish-green appearance. Upon opening the bags, a characteristic mildly acidic and mellow aroma was observed. The FF demonstrated excellent organoleptic and physical characteristics, featuring a non-adhesive texture, optimal moisture content, favorable flowability, and uniform particle distribution. Fermentation quality analysis indicated that lactic acid was the predominant fermentation product (3.74%), with lower concentrations of acetic acid (0.56%) and butyric acid (0.07%), while propionic acid was not detected. Microbiological analysis showed that the viable count of lactic acid bacteria reached 8.85 ± 0.10 log_10_ CFU/g, whereas *Saccharomyces cerevisiae* was not detected. Analysis of mycotoxins showed that AFB_1_, OTA, and ZEN were not detected, whereas DON was detected at 0.30 mg/kg DM, which was below the maximum permissible level specified in the Chinese Feed Hygiene Standard (GB 13078-2017) [27]. The nutritional composition analyzed for the FF is detailed in Table 3.

### 3.2. Growth Performance

Table 4 indicates that there were no notable differences in growth performance observed between the CON group and the group receiving 20% FF (*p* > 0.05).

### 3.3. Apparent Total Tract Digestibility of Nutrients

The dietary treatments had no significant impact on the digestibility (*p* > 0.05; Table 5).

### 3.4. Carcass Characteristics

Table 6 indicates that there were no notable differences in carcass trait parameters when comparing the CON group to the group that received 20% FF (*p* > 0.05).

### 3.5. Meat Quality Parameters

Table 7 illustrates that there were no notable differences in the objective meat color coordinates (L*, a*, and b*) when comparing the CON group with the 20% FF group (*p* > 0.05). Nevertheless, the addition of 20% FF notably decreased the concentration of muscular MDA (*p* = 0.009) and increased the activity of GSH-Px (*p* = 0.009).

Based on the data presented in Table 8, including 20% FF in the diet did not significantly affect muscle fatty acid levels relative to the CON group (*p* > 0.05).

## 4. Discussion

The findings from this research showed that substituting 20% of the basal diet with FF did not result in notable differences in growth performance in comparison to the CON group. Special attention should be paid to the fact that the actual concentrations of dietary gross energy and crude protein in the group receiving 20% FF were slightly lower than those observed in the CON group. In monogastric nutrition, reduced dietary nutrient density typically compromises growth rates [28]; however, no reduction in growth performance was observed in this study. This consistency might be somewhat linked to the “pre-digestion” effects of the fermentation process on the forage matrix. Anaerobic microbial fermentation effectively degraded recalcitrant plant cell walls and anti-nutritional factors while concurrently synthesizing substantial quantities of functional substances [8]. These bioactive metabolites are instrumental in ameliorating the intestinal microenvironment, stimulating digestive enzyme activity, and facilitating nutrient absorption, thereby optimizing overall nutrient utilization [29,30,31]. Consistent with this, Zhu et al. indicated that incorporating 50% fermented alfalfa into swine diets notably enhanced the utilization of crude fiber while maintaining growth performance even at a relatively high substitution level [9]. Liu et al. observed that incorporating 5% and 10% fermented mixed feed into the diet resulted in enhanced average daily gain (ADG) among finishing gilts, while no significant effects were detected regarding the performance of barrows [11]. Consequently, dietary inclusion of 20% FF maintained the growth performance of finishing pigs without adversely affecting productive performance, demonstrating the feasibility of partially replacing the basal diet with FF under the experimental conditions of this study.

Apparent nutrient digestibility serves as a critical parameter for evaluating the in vivo utilization efficiency of dietary nutrients. The present findings indicate that the 20% FF treatment did not significantly impair the apparent digestibility of GE, DM, CP, or ash. This suggests that, at optimal inclusion levels, FF does not compromise the digestive capacity of finishing pigs for macronutrients. This aligns with several recent studies evaluating fermented forages and fibrous substrates in swine nutrition. Cheng et al. indicated that the inclusion of compound protein derived from enzymolysis–fermentation in finishing diets did not result in any notable differences in the apparent digestibility when compared to the control diets [26]. According to Liu et al., the inclusion of 5% to 15% fermented mulberry (FM) in the basal diets of finishing pigs did not change their apparent digestibility [32]. Moreover, systematic meta-analyses have concluded that fermented feeds, when compared against conventional diets, do not significantly disrupt the total tract digestibility of primary nutrients, a stability that is particularly pronounced in mature swine [33].

The potential improvement in GE digestibility may be associated with the favorable fermentation characteristics of the FF, which are demonstrated by its low pH, elevated levels of lactic acid, and a high count of active lactic acid bacteria. Fermentation-derived organic acids lower the pH of gastrointestinal digesta, which in turn stimulates endogenous enzyme activity and optimizes the nutrient absorptive milieu [34]. Concurrently, alterations in the physicochemical properties of dietary fiber during fermentation are intimately correlated with enhanced digestibility and energetic yield; controlled fermentation thus mitigates the anti-nutritional effects typically associated with high-fiber diets in monogastric animals [35]. Consequently, the dual benefits of “microecological optimization” and “fiber matrix disruption” induced by fermentation effectively offset the potential negative impacts of fibrous substrates on digestibility, ensuring robust nutrient utilization.

Carcass traits are paramount indicators that dictate production efficiency and the economic value of swine. Carcass characteristics are predominantly governed by genetic potential and exhibit relative resilience against short-to-medium-term or moderate nutritional interventions, provided that severe nutrient deficiencies are avoided [36]. The present findings confirmed that 20% FF exerted no adverse impacts on dressing percentage, lean meat yield, backfat thickness, or other related traits, suggesting that energy partitioning between adipose and lean tissue deposition remained undisturbed at this inclusion level. These observations are corroborated by existing literature. Zhu et al. and Han et al. both found that the addition of moderate amounts of fiber-rich fermented feeds does not adversely affect the carcass composition of finishing pigs [9,37]. Furthermore, in a trial involving 144 finishing pigs, diets supplemented with 5% and 10% fermented mixed feed yielded carcass traits statistically indistinguishable from the CON group, reaffirming that microbial fermented feed, within rational limits, does not impair critical carcass parameters [11].

Despite the slight reduction in dietary nutrient density in the 20% FF group, the fermentation-induced enhancement of nutrient bioavailability underscores the robust adaptive metabolic capacity of finishing pigs to such nutritional modulations. This adaptability is likely facilitated by the fermentative breakdown of complex physical fiber structures, enhancing their hindgut fermentability and thus averting the energetic dilution effect typical of high-fiber diets. Furthermore, this phenomenon may also be associated with organic acids and specific microbial metabolites generated during fermentation; these beneficial substances could potentially optimize the intestinal microecology, promote systemic nutrient absorption, and modulate lipid metabolic pathways, ultimately preserving ideal backfat thickness and lean tissue ratios [35].

Meat color serves as a primary determinant of pork sensory quality and consumer purchasing intent, with its stability critically dependent on the oxidative state of myoglobin. The current investigation revealed that incorporating 20% FF exerted no significant effects on the objective meat color coordinates of the *Longissimus thoracis et lumborum* muscle, suggesting that this inclusion strategy successfully preserves the visual appeal of the pork while achieving feed cost reductions. Notably, despite the static meat color parameters, biochemical indices indicative of muscular antioxidant capacity exhibited marked improvements. Specifically, the 20% FF treatment significantly attenuated intramuscular MDA concentrations while concurrently elevating GSH-Px activity, alongside a discernible trend toward increased SOD activity. These findings robustly confirm the efficacy of fermented forage-based mixed feed in bolstering the antioxidant potential of pork, a conclusion highly congruent with prior research evaluating diverse plant-based fermented feeds. A study conducted by Ma and colleagues indicated that incorporating fermented pine needles into the diet during the fattening phase led to a notable rise in the mRNA expression levels of genes related to antioxidants in both serum and muscle tissue, as well as a decrease in the MDA content in muscle [12]. Analogously, Xie et al. observed that fermented soybean meal enhanced systemic antioxidant capacity and mitigated lipid peroxidation (MDA content) in pork [38].

The mechanism underlying the FF-mediated enhancement of muscular antioxidant capacity may be partially associated with bioactive compounds generated during microbial fermentation. Previous studies have reported that microbial fermentation can generate or increase various bioactive compounds, including organic acids, microbial secondary metabolites, flavonoids, and phenolic compounds. These natural antioxidants effectively scavenge reactive oxygen species and augment the activities of endogenous antioxidant enzymes, thereby collectively enhancing the antioxidant status of the animals [39].

The fatty acid profile that remained consistent in this study could be due to a variety of factors. First, FF may have contained relatively low concentrations of fatty acid precursors, such as α-linolenic acid, limiting their deposition in muscle tissue. Second, the dietary inclusion level (20%) may not have been sufficient to induce measurable alterations in intramuscular lipid composition. Furthermore, the 56-day duration of the feeding period might have been too short for the dietary intervention to produce noticeable alterations in the composition of muscle fatty acids. Although no significant differences were observed in fatty acid composition, incorporating 20% FF did not negatively influence the overall lipid profile of pork based on the conditions observed in this study.

## 5. Conclusions

This research thoroughly assessed the effectiveness of utilizing fermented forage-based mixed feed in the diets of finishing pigs. The findings suggested that substituting 20% FF for the basal diet during the finishing phase had no negative impact on the pigs’ growth performance, apparent nutrient digestibility, or carcass characteristics. Concurrently, the inclusion of FF significantly increased the activity of antioxidant-related enzymes in the pork, actively contributing to the improvement of its oxidative stability. In conclusion, under the present experimental conditions, FF serves as a viable feed resource for replacing a portion of the basal diet in finishing pigs.

## Figures and Tables

**Table 1:** Proportion of fermented forage-based mixed feed.

Ingredients	Weight
Forage powder (kg)	430
Wheat bran (kg)	275
Cornmeal (kg)	220
Soybean meal (kg)	55
Microbial inoculum (kg)	20
Total (kg)	1000

**Table 2:** The composition of the basal diet and nutrient levels (%, as-fed basis).

Ingredients	CON	20% FF
Corn	66.00	52.25
Soybean meal, 43%	15.00	11.88
Wheat bran	15.00	11.87
FF	0.00	20.00
Premix ^1^	4.00	4.00
Total	100.00	100.00

^1^ Dietary premix provided per kilogram includes 35.2 mg of vitamin A; 4 mg of vitamin B1; 12 mg of vitamin B2; 8.32 mg of vitamin B6; 4.8 mg of vitamin B12; 7.68 mg of vitamin D3; 128 mg of vitamin E; 8.16 mg of vitamin K3; 110 mg of zinc (as ZnSO_4_·H_2_O); 125 mg of copper (as CuSO_4_·5H_2_O); 0.19 mg of selenium (as Na_2_SeO_3_); 171 mg of iron (as FeSO_4_·H_2_O); 0.19 mg of cobalt (as CoCl_2_); 42.31 mg of manganese (as MnSO_4_·H_2_O); and 0.54 mg of iodine (as Ca(IO_3_)_2_).

**Table 3:** Calculated nutritional levels of fermented forage-based mixed feed and experimental diets (as-fed basis).

Nutritional Levels	Treatments ^1^		
	FF	CON	20% FF
GE (MJ/kg)	14.65	15.88	14.93
DM (%)	58.97	87.73	81.77
CP (%)	9.77	14.68	13.80
EE (%)	2.51	1.03	0.83
CF (%)	15.83	3.09	3.93
NDF (%)	37.28	4.01	5.06
ADF (%)	11.98	10.94	13.15
Ash (%)	4.71	4.59	4.30
AIA (%)	0.45	0.33	0.37
Ca (%)	0.17	0.60	0.50
P (%)	0.28	0.55	0.47
Lys (%)	0.50	0.80	0.70
Met (%)	0.08	0.22	0.19
Thr (%)	0.33	0.50	0.44
Trp (%)	0.06	0.14	0.12
Lactic acid	3.74	-	-
Acetic acid	0.56	-	-
Propionic acid	ND ^2^	-	-
Butyric acid	0.07	-	-
pH	4.20	-	-

^1^ FF: fermented forage-based mixed feed; CON: basal diet; 20% FF: diet with 20% of the basal diet replaced by fermented forage-based mixed feed. ^2^ ND: not detected.

**Table 4:** Impact of fermented forage-based mixed feed on growth performance.

Performance Parameters	Treatments		SEM ^1^	*p*-Value
	CON	20% FF		
BW (kg)				
Day 0	65.74	65.85	0.93	0.933
Day 28	93.46	93.09	1.91	0.898
Day 56	118.34	118.52	1.87	0.953
ADG (g)				
Day 0–28	990	973	49	0.814
Day 28–56	889	908	30	0.696
Day 0–56	939	940	24	0.978
ADFI (g)				
Day 0–28	2627	2603	45	0.714
Day 28–56	3320	3364	65	0.700
Day 0–56	2974	2983	46	0.889
F/G				
Day 0–28	2.67	2.69	0.10	0.884
Day 28–56	3.74	3.71	0.07	0.714
Day 0–56	3.17	3.18	0.06	0.871

^1^ SEM is the standard error of the mean. *n* = 4 per treatment group.

**Table 5:** Impact of fermented forage-based mixed feed on apparent digestibility.

Apparent Digestibility Parameters	Treatments		SEM	*p*-Value
	CON	20% FF		
GE (%)	65.73	72.96	2.66	0.110
DM (%)	88.14	89.25	0.67	0.289
CP (%)	87.18	86.66	1.10	0.752
Ash (%)	59.78	58.90	2.40	0.804

*n* = 4 per treatment group.

**Table 6:** Impact of fermented forage-based mixed feed on carcass traits.

Carcass Parameters	Treatments		SEM	*p*-Value
	CON	20% FF		
Dressing percentage (%)	75.96	73.53	1.11	0.174
Lean meat percentage (%)	58.65	58.52	1.19	0.947
Fat percentage (%)	19.98	19.76	1.33	0.920
Skin percentage (%)	5.98	5.83	0.16	0.552
Bone percentage (%)	13.25	13.46	0.32	0.653
Ham and buttock percentage (%)	32.19	32.24	0.63	0.954
Backfat thickness (mm)	23.63	23.55	0.75	0.945

*n* = 4 per treatment group.

**Table 7:** Impact of fermented forage-based mixed feed on muscle color and antioxidant enzymes.

Meat Quality Parameters	Treatments		SEM	*p*-Value
	CON	20% FF		
L*	45.50	43.36	1.86	0.447
a*	15.14	15.33	0.66	0.875
b*	9.11	10.59	0.87	0.332
CAT (U/mg prot)	2.16	2.27	0.22	0.744
SOD (U/g prot)	29.38	34.09	2.29	0.197
GSH-Px (U/mg prot)	1.76	2.58	0.14	0.009
MDA (nmol/g prot)	10.88	8.40	0.44	0.009

*n* = 4 per treatment group.

**Table 8:** Impact of fermented forage-based mixed feed on muscle fatty acids (as-fresh basis).

Meat Fatty Acid Composition	Treatments		SEM	*p*-Value
	CON	20% FF		
C14:0 (g/kg)	0.73	0.84	0.13	0.572
C15:0 (g/kg))	0.07	0.06	0.03	0.751
C16:0 (g/kg)	13.46	15.49	2.33	0.561
C16:1 (g/kg)	1.44	1.65	0.32	0.673
C17:0 (g/kg)	0.04	0.07	0.02	0.427
C17:1 (g/kg)	0.08	0.11	0.03	0.529
C18:0 (g/kg)	7.79	8.96	1.34	0.557
C18:1n9c (g/kg)	23.73	25.33	3.96	0.785
C18:2n6c (g/kg)	5.57	5.98	0.83	0.754
C20:0 (g/kg)	0.07	0.11	0.03	0.406
C20:1 (g/kg)	0.49	0.57	0.09	0.549
C18:3n3 (g/kg)	0.22	0.23	0.04	0.812
C20:2 (g/kg)	0.24	0.29	0.05	0.533
C22:0 (g/kg)	0.03	0.05	0.01	0.173
C20:4n6 (g/kg)	0.50	0.56	0.03	0.273

*n* = 4 per treatment group.

## Data Availability

The original contributions presented in this study are included in the article. Further inquiries can be directed to the corresponding authors.
